# Psychometric evaluation of a self-reported physical activity questionnarie used in the pilot phase of the AZAR Cohort Study

**DOI:** 10.15171/hpp.2016.24

**Published:** 2016-08-10

**Authors:** Maryam Mirzaei, Mohammad Asghari-Jafarabadi, Nayyreh Amini-Sani, Fatemeh Bakhtari-Aghdam, Saeed Dastgiri

**Affiliations:** ^1^Department of Biostatistics & Epidemiology, Tabriz University of Medical Science, Tabriz, Iran; ^2^Road Traffic Injury Research Center, Tabriz University of Medical Science, Tabriz, Iran; ^3^Department of Health Education & Promotion, Tabriz University of Medical Science, Tabriz, Iran; ^4^Tabriz Health Services Management Research Centre,Tabriz University of Medical Sciences, Tabriz, Iran

**Keywords:** Physical activity, Validity, Reliability, Questionnaire

## Abstract

**Background:** The purpose of this study was to assess the psychometric properties of a self-reported physical activity (PA) questionnaire based on data from the pilot phase of the AZAR Cohort Study.

**Methods:** In this cross-sectional study, all 35-70 years old people living in Khameneh, a city in East Azarbaijan, Iran were invited to take part in the pilot phase of the AZAR Cohort Study. A total of 952 people completed the self-reported PA questionnaire and the International Physical Activity Questionnaire (IPAQ). Construct validity was evaluated by exploratory and confirmatory factor analyses (EFA and CFA). Spearman’s correlation coefficient between the scores of the two instruments was used to examine the concurrent validity. Reliability was measured using intraclass correlation coefficient (ICC) and Cronbach’s alpha coefficient.

**Results:** In EFA applying principal component analysis with varimax rotation, four factors were identified including recreational leisure time (variance = 52.73%), sedentary leisure time (variance = 38.68%), household/gardening work (variance = 38.66%), and occupation work (variance = 12.67%). The extracted factors were also supported by the CFA (CFI = 0.98, GFI =0.936, RMSEA=0.057). The results indicated moderate concurrent validity (ρ = 0.62, P < 0.001). ICC and Cronbach’s alpha were 0.59 and 0.7, respectively.

**Conclusion:** These results showed acceptable and moderate psychometric properties for the self-reported PA questionnaire to assess PA in this population-based study.

## Introduction

The link between physical activity (PA) and health status has been evaluated in various epidemiological studies. Physical inactivity poses a behavioral risk factor for some types of non-communicable diseases (NCDs) including cardiovascular diseases, stroke, high blood pressure, non–insulin-dependent diabetes mellitus, osteoporosis, and certain types of malignancies.^1-5^ Nevertheless, only a small proportion of individuals gets sufficiently adequate PA.^6^

PA measurement is hard to quantify due to its complex nature.^7,8^ In data collection at the population level, selecting appropriate and precise estimation method to measure PA as a variable is a challenging task for investigators.^5-9^ In comparison, a variety of methods have been used to assess PA, such as self-reports (interviews, diaries, and questionnaires) and doubly-labeled water at population-based studies. Self-reported PA questionnaires are usually chosen as the most feasible methods to assess PA. As such questionnaires are inexpensive, brief, and have general acceptance, they can be applied to measure the frequency, duration, and intensity of PA.^10,11^

The AZAR Cohort Study, initiated in 2014, is a population-based follow-up study conducted in East Azerbaijan province, Iran. Investigating the level of PA and its effects on the health status of the population in this prospective cohort study has been considered as a crucial task. Hence, a valid and reliable questionnaire was required to determine the habitual PA of the people in this large-scale epidemiological study.^5^ In general, several questionnaires have been validated to assess PA among adults, but each instrument has some disadvantages. ^8^

In order to conduct this cohort study, there was a need for a scale developed to measure PA by questionnaire only, which particularly designed for a large epidemiological study.^12^ This self-reported PA questionnaire was designed, developed, and validated by Aadahl and Jorgensen in 2003, and it is a PA scale with a simple usage to measure the level of PA among sedentary adults. This questionnaire was previously modified and validated among Danish adults.^12,13^ In different settings, the accuracy of the self-reported PA questionnaires depends on the diversity of the sociocultural and ethnic context. Therefore, in order to assure an accurate measurement of PA level in a specific population there is a need to examine the validation of the tool being used for the study.^4^

Although the Persian translation of this questionnaire was previously used for the Childhood & Adolescence Surveillance and Prevention of Adult Non-communicable Diseases (CASPIAN) study,^14,15^ among adult population, no validated Iranian version has been reported to date.

Previous studies reported the concurrent validity of this self-report PA questionnaire.^12,13^ In those studies the findings of the questionnaire were compared with accelerometer, PA dairy questionnaire and Vo_2max_. Additional studies are needed to validate it among other target populations. In addition, the factor structure of the Persian version of the questionnaire needs to be established. Searching the literature no published study was found on the factor structure of this PA scale among Iranian adult population. The present study aimed to assess the validity of this self-reported PA questionnaire by examining the construct, discriminate and concurrent validities. Temporal reliability and internal consistency were also examined. Therefore, the primary objectives of this study were to explore the dimensionality and evaluate psychometric properties (validity and reliability) of the self-report PA questionnaire in the pilot phase of the AZAR Cohort Study conducted on 35-70 years old population in Khameneh in 2014

## Materials and Methods

### Sample and data collection

The AZAR Cohort Study, a state-level of a nationwide cohort study (Persian cohort, http://persiancohort.com) in Iran, is a longitudinal study assessing risk factors related to the most prevalent NCD in East Azarbaijan province. This cohort study has been conducted by Tabriz University of Medical Sciences in Shabestar – a county located in East Azarbaijan province. All the invited people for taking part in this study were 35 to 70 years old and met the inclusion criteria (the permanent resident of this city, ability to response to the questions, Iranian originality). Exclusion criteria were refusal to participate in the study, being in travel out of the area during the study period and being with mental and physical disabilities.

The pilot phase of the AZAR Cohort Study was conducted in Khameneh, a small town in Shabestar county between October 2014 and January 2015. The target participants for this investigation were elected from the framework of the pilot phase of the study which included 952 respondents (35-70 years, mean: 49.84, standard deviation: 8.82). During the first questionnaire survey (participation rate = 82%), the participants took part in in-site interviews by trained interviewers. The baseline socio-demographic characteristics including age, education, occupation, nutritional habits, medical history, and anthropometric data such as height and weight, as well as the self-report PA were investigated.

### Physical activity instrument

In order to measure PA in this large sample, the validated self-reported PA questionnaire as a scale to assess PA was utilized, which has previously been shown to be valid and reliable in the Danish population.^13^ This classified self-reported PA, consisted of 23 items based on nine ranges that have different metabolic equivalent (MET) activities (from sleep/rest [0.9 METs] to high-intensity PA [>6 METs]). The participants had to report all domains of their PA, such as occupational PA (PA at work), recreational PA (leisure time PA), and exercise activity on an average weekday; in all domains, the amount of time spent on sedentary behaviors was also assessed. To estimate the MET-time scores, the times engaging in scales were multiplied by an estimate of the METs of the reported activity. Also, to achieve the same scale, MET-h was calculated from the MET-min in International Physical Activity Questionnaire (IPAQ), by adding the MET-time from all weekday and dividing by 60 minutes.

### Study procedures for psychometric tests

The process of the translation and cultural adaptation was performed in previous studies by Kelishadi et al.^14,15^ Thus, the content validity using a qualitative manner was assessed by cohort investigators before the commencement of this study and some minor changes were made to revise the wording and structure of some sentences. The psychometric properties of the questionnaire included three specific methodological steps:

*First step:* Reliability was evaluated by determining internal consistency (Cronbach’s α) and temporal stability which was assessed over a period of two weeks of test-retesting. In the first step of this process, 50 participants were recruited based on a list of random numbers for testing the reproducibility of the questionnaire at two time points.

*Second step:* Concurrent form of criterion validity for the self-reported PA questionnaire was evaluated by comparing its total score with IPAQ – as a criterion measure. IPAQ is known as an accurate scale with a confirmed validity and reliability in an Iranian population.^16^ To answer the research questions concerning the association between the self-reported PA questionnaire and the IPAQ, a subset (n = 50) of the participants was also asked to complete the IPAQ. In addition, the Bland-Altman plot, 95% limits of agreement, was utilized to show graphically the agreement between the self-report PA and IPAQ.

*Third step:* In order to determine the underlined structure of the items and test the hypothesized structures, the construct validity was determined by exploratory factor analysis (EFA) and confirmatory factor analysis (CFA). Furthermore, the construct validity was evaluated by performing the known groups’ comparison - as an additional approach to establishing construct validity. It was hypothesized that known groups (gender, education, and occupation subgroups) would report different total scores. The questionnaire was considered valid based on these criteria.

### Statistical analyses

Temporal stability and internal consistency were measured using intraclass correlation coefficient (ICC) and Cronbach’s alpha coefficient, respectively. In this study, the ICC and Cronbach’s alpha more than 0.7 were considered as acceptable reliabilities.^17,18^ In order to conduct the known group analyses and the hypothesis that the total scores would be significantly different between the subgroups, the Kruskal-Wallis H test and the Mann–Whitney U test were performed to compare the subgroups.

EFA and CFA approaches were implemented to identify the factor structure of the questionnaire. In the first step, principal component analysis (PCA) was used to extract the factors, with the assumption of the abnormality of the data distribution and the optimality of the procedure. Also, due to the independency of the factors, the varimax orthogonal rotation was applied in the EFA.^19,20^ Factor-item loading values were considered acceptable to offer an item to a factor if the value was equal to or greater than 0.20. The significant eigenvalues was considered equal to or greater than1.0. The Kaiser-Meyer-Olkin (KMO) method and Bartlett’s test of sphericity were performed to test the sampling adequacy. In the second step, the CFA model using the robust maximum likelihood was used to estimate model parameter. The absolute fit of the model to the data was evaluated using the χ^2^ statistic, root mean square error of approximation (RMSEA), goodness-of-fit index (GFI), adjusted goodness-of-fit index (AGFI), and the comparative fit index (CFI). Values of the GFI, AGFI, and CFI greater than 0.90, and the RMSEA value below 0.08 was acceptable as a good model fit.^21^All data analyses were performed by SPSS 23.0 (Chicago, IL, USA), also other complementary software according to the objectives presented in the related sections and the statistical significance level was set at *P*<0.05.

## Results

### General characteristics of the study participants

Table 1 shows the characteristics of the total sample in the EFA (n = 952) and the sub-sample used to determine CFA (n = 572). The age range of the total sample was 35 to 70 years with a mean of 49.84 (SD = 8.82) years. The most of the participants were married (91.7%), 11.5% were with no formal education, and 50.7% were employed. In addition, the body mass index (BMI) value for 42.7% of the participants was in the range of 25-30 (overweight).

### Temporal stability and internal consistency

The analysis of test-retest reliability with the method of ICC showed moderate temporal stability for self-reported PA items at two time points (0.59; 95% CI: 0.20–0.74). It should be noted that the log transformation improved normality and these values were used throughout the analysis. The internal consistency (coefficient Cronbach’s α) for the scale was 0.7, which indicated satisfactory internal consistency.

### Factorial (construct) validity

EFA was conducted in the scale base to identify the factor model using all the observations. Based on the origin structure and the preliminary analyses of the items, it was found that the extracted factors may be divided into four sub-scales as detailed below:

1. Inactive leisure time, 5 items (PB1-PB2-PA1-PA2-PA3)
2. Household/gardening work, 3 items (PD1-PE1-PG2)
3. Occupation work, 11 items (PC1-PC2-PC3-PD2-PF4-PH1-PE2-PG1-PF2-PF3-PG3).

More specifically, the dimension reduction process was implemented for all the domains, separately, to identify underling potential factor (sub-scales). Table 2 shows the results of the scale based test of the item convergence validity. The final model found to be with four factors and 21 items (two items did not load on any factor [factor loadings < 0.2] and was removed): Factor 1 with 4 items and 2 sub-factors (variance = 52.73%), factor 2 with 6 items and 2 sub-factors (variance = 38.68%), factor 3 with 3 items (variance = 38.66%), and factor 4 with 10 items (variance = 12.67%). This model indicated that the extracted factors were suitable for the factor model in the observed dataset. The extracted sub-factors were named as sports (2 items), walking & bicycling (2 items), sitting (3 items), and sleep (3 items). The other recognized factors were given the same names as the basic factors of the underlying domains. In addition, the eigenvalues of all the domains were more than 1. The factor analysis results showed the value of the KMO measure of the sampling adequacy to be 0.69, and Bartlett’s test of sphericity showed the adequacy of the model (*P*<0.001).

**Figure 1:**
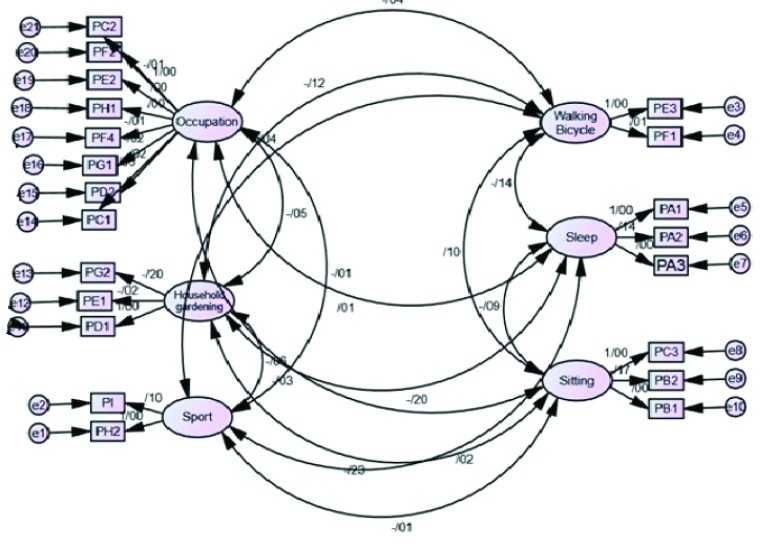
Confirmatory factor analysis.

In order to achieve a CFA model with a good external validity, it is highly recommended to perform the CFA in a random subsample (a random sample drown from the main sample is normally satisfied).^20^ To do so, the CFA was conducted on the 21 items of the questionnaire with AMOS 23.0 software to test the fit of the final four-factor model. Sixty percent of the participants were considered as the sub-sample; through random sampling the data of 60% out of all the participants in the SPSS software were included in the CFA analysis. CFA supported the four-factor structure and displayed appropriate good fit to the data (χ2 [163] = 462.139, *P*<0.001; CFI = 0.98; GFI = 0.936; AGFI = 0.90; RMSEA [90% CI] = 0.057 [(0.51-0.063]). Moreover, all the standardized coefficients in the factor showed moderate correlations between the latent factors (Figure 1).

### Concurrent validity 

Spearman’s correlation coefficient between the scores (MET-time) of the self-report PA questionnaire and the IPAQ showed positive and moderate-to-good correlations (r = 0.62) between the factors, which was statistically significant (*P*<0.001). Also, the Bland-Altman plot indicated moderate agreement between the two instruments (Figure 1). In the Bland-Altman plot, the mean difference was -0.02 with wide 95% limits of agreement (-0.2 to 0.25), but four out of 50 values (8%) were outside the 95% limits of agreement.

### Known group’s analyses

In the known group’s analysis, the item-discriminant validity aspect was tested by the relative test. Significant differences in MET-time score were observed only by job, marital status, and education subgroups. As it was expected, the difference was found in the MET-time score between employed and unemployed. The difference was statistically significant (*P*<0.05). Unemployed group had lower MET-time score. Also, as hypothesized, the Kruskal-Wallis H test revealed significant difference in the total score in MET-time by education and marital status (Table 3).

**Table 1:** General characteristics of the study participants

**Variables**	**Total sample for EFA (n=952)**	**Subsample for CFA (n=572)**
	**No. (%)**	**No. (%)**
Gender		
Male	440 (46.1)	256 (44.8)
Female	514 (53.9)	316 (55.2)
Age (years)		
35-45	313 (32.8)	202 (35.3)
45-55	385 (40.4)	227 (39.7)
55-65	210 (22)	120 (21.0)
≥65	46 (4.8)	23 (4.0)
Marital status		
Unmarried	24 (2.5)	17 (3.0)
Married	875 (91.7)	525 (91.8)
Divorce/widow	55 (5.8)	30 (5.2)
Educational level		
No formal education	114 (11.9)	66 (11.5)
Elementary	281 (29.5)	170 (29.7)
Middle school	177 (18.6)	113 (19.8)
High school	202 (21.2)	117 (20.5)
College/ university and above	180 (18.9)	106 (18.5)
Occupation status		
Employed	484 (50.7)	293 (51.2)
None	470 (49.3)	279 (48.4)
BMI (kg/m^2^)		
Underweight (BMI <18.5)	10 (1.0)	7 (1.2)
Normal weight (18.5-25)	241 (25.3)	135 (23.6)
Overweight (25-30)	407 (42.7)	235 (41.1)
Obese (BMI ≥30)	296 (31.0)	195 (34.1)

Abbreviations: BMI, body mass index; EFA, exploratory factor analysis; CFA, confirmatory factor analysis.

**Table 2:** Exploratory factor loading (principal axis factoring extraction with varimax rotation) of the questionnaire items^a^

**Factor**	**Subfactor**	**Item**	**Loading**
Factor 1. Recreational leisure time	Sport	PH2	0.729
		PI	0.710
	Commuting (Walking/bicycle)	PE3	0.781
		PF1	0.581
Factor 2. Sedentary leisure time	Sitting	PC3	0.769
		PB2	-0.565
		PB1	0.390
	Sleep	PA1	0.728
		PA2	0.617
		PA3	0.330
Factor 3. Household/gardening work		PG2	-0.687
		PD1	0.683
		PE1	0.471
Factor 4. Occupation work		PC1	-0.720
		PD2	0.504
		PG1	0.467
		PF4	0.338
		PH1	0.221
		PE2	0.231
		PF2	0.212
		PC2	-0.211

^a^All loadings above 0.20 are presented; the negative values show indirect relation between an item and a scale.

**Table 3:** PA profile (MET-time scores) of the study participants

**Known groups**	**Median (P25 to P75) N = 952**	***P *** **value**
Total PA	35.25 (33.11-38.12)	
Employment		<0.001^a^
Unemployment	34.85 (32.91-37.45)	
Employment	35.63 (33.35-38.90)	
Marital status		0.026^b^
Unmarried	34.15 (32.‏22-36.‏70)	
Married	35.38 (33.18-38.26)	
Divorce/widow	34.53 (31.40-36.70)	
Level of education		<0.001^b^
No formal education	35.13 (33-38.70)	
Elementary	35.83 (33.93-38.70)	
Middle school	34.98 (33.05-37.80)	
High school	35.48 (32.85-38.45)	
College/ university and above	34.43 (32.38-36.65)	
BMI (kg/m^2^)		0.527^b^
<18.5	36.18 (34.15-39.60)	
18.5-25	35.53 (33.43-38.26)	
25-30	35.21 (33.08-38.15)	
>30	35.19 (32.93-37.84)	
Gender		0.285^a^
Male	35.28 (32.83-39.14)	
Female	35.25 (33.30-37.63)	

Abbreviations: BMI, body mass index; PA, Physical activity; MET, metabolic equivalent. Median (Percentile 25 to Percentile 75) was reported. ^a^Mann-Whiteny U test; ^b^Kruskal-Wallis H test.

## Discussion

Insufficient PA is considered as a behavioral risk factor for NCDs. Without a valid instrument, the associations between PA and health status may not be accurately evaluated and identified.^22,23^ The present study sought to determine whether the selected self-reported PA questionnaire was a valid scale to assess PA patterns among the adults elected for the AZAR Cohort Study. From the results of the present study confirmed the internal consistency of the scale. Moreover, moderate temporal stability of the questionnaire during two separate occasions (correlation coefficient = 0.60) was found. These findings were in line with those obtained in the study that assessed the PA among Iranian young adults (correlation coefficient = 0.87).^14^

Although the ICC value showed moderate temporal stability, the 95% CI was wide for the scores. This wide 95% CI for the ICC value could be due to the fact that the PA has a multinomial nature and it is not a stable behavior.^7,8^ However, it does not seem that the actual changes in the PA pattern of the subjects occurred during 2-weeks interval between the test-retest. The moderate coefficient of the correlation between the two occasions provided evidence for temporal validity of the self-reported PA questionnaire.

This is the first investigation to address the EFA and CFA approach of the self-reported PA questionnaire. The results confirmed the factorial structure of the questionnaire in a sample of 952 adults. The sample size was adequate for factor analysis in the present study as the proportion of the sample size was based on more than 5 participants per item.^24^

The findings of the present study suggested that the questionnaire might have a four factors structure for the instrument, including recreational leisure time, sedentary leisure time, household/gardening work, and occupation work. The results showed that the factor loadings of the items PG3 and PF3 were less than 0.2. As, the considered cut-off value to retain an item in the scale was 0.2, these items were not included in the final model. Also, the CFA provided evidence to support the factor structure represented by these items. Therefore, the questionnaire assessed PA in four factors (domains) and four sub-factors with 21 items. A concern in the analysis of this instrument was its construct validity which had not been investigated previously. So, the identified model should be further assessed. However, the preliminary factor structure found in the present study was not different from those found in the original domains. Previously, some validation studies on the questionnaire have been conducted in Denmark and Iran, but the demographics and geographic contexts of the studies were different.^13,14^

In the present study, the IPAQ, as a subjective measure, was considered as a criterion measure for concurrent validity. In consistent with the findings of the prior research in Denmark,^13^ a significant but not so strong correlation was found between the two self-reported questionnaires (r = 0.62, *P*<0.001). However the significant moderate correlation confirmed the concurrent validity of the PA questionnaire. Concurrent validity of the questionnaire was assessed among Danish adults by Aadahl and Jorgensen in 2003. They found a high correlation between the scale and a PA dairy questionnaire (r = 0.74) and a poor correlation between the scale and an accelerometer (r = 0.20, *P* = 0.04); so the self-reported PA scale was approved as a valid instrument to assess the PA among the adult with sedentary to moderately active populations.^13^ In a study to validate the self-report PA questionnaire against maximal oxygen uptake (Vo_2max_ testing), correlation between the two scales was assessed and it was found that the activity scale had an acceptable validity.^12^ In this study, the total amount of PA was not significantly associated with Vo_2max_ (r^2^= 0.69, *P* = 0.098), but the amount of daily vigorous intensity PA and Vo_2max_ had a strong and significant association (r^2^= 0.76, *P*<0.001).^12^ As a matter for validation, using subjective methods to assess criterion validity can be considered as a limitation for the present study. The PA questionnaires as an objective measure are prone to recall and desirability biases. Hence, the result of subjective methods should be paid attention in terms of misclassification while assessing the PA habits.^12,25,26^

**Figure 2:**
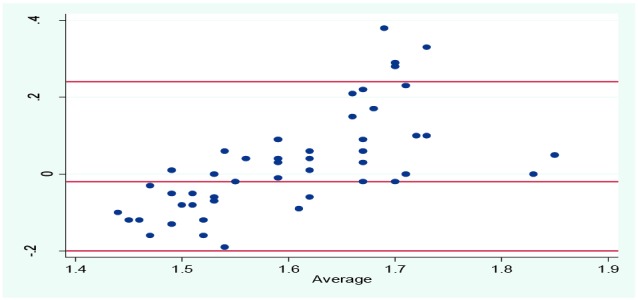
Bland-Altman plot: Difference in MET-time scores from the activity scale versus MET-time scores from the IPAQ (y-axis), plotted against the average MET-time scores of the two measures (x-axis). The central horizontal line represents the mean difference and the flanking lines represent the 95% limits of agreement. Mean difference: -0.02±2, SD = -0.2 to 0.24.

The Bland-Altman plot was used to verify the agreement between the two questionnaires, but a non-constant bias was observed over the whole range of the instruments. Therefore, as recommended by Bland-Altman, log transformation approach was applied in the present study.^27^Nevertheless this method could not improve the agreement between the two scales. As shown in the Bland-Altman plot (Figure 2), the discrepancy between the two scales was obvious in lower and higher values (a trend line for bias) and it is increased with increasing/decreasing total MET-time values. This discrepancy infers that activities with moderate intensity are being measured more accurately compared to the light/vigorous intensity activities. The results of our study are consistent with those found in the previous study.^13^

In another aspect of the results of the present study, one may note that the mean difference between the two methods was small, but the 95% limits of the agreement in the mean scores of MET-time values between the two self-reported questionnaires were wide and affected by four outliers. This would be an evidence for bias between the activity scales and, therefore, the agreement between the instruments may not be a gratifying result. Although the objective methods may provide more accurate information, it is not feasible to be used in population-base surveys.^10,11^ Additional validation studies of the self-reported PA questionnaire against objective methods is recommended in pilot studies with smaller sample size. In the rest of the cohort profile, exploring the predictive validity of the self-reported PA questionnaire is suggested. This exploration may be conducted through examining the relationships among self-reported time spent to PA and several health outcomes related to chronic diseases, such as blood pressure (BP) and High-density lipoprotein (HDL) and low-density lipoprotein (LDL) cholesterol.

As another finding, when the known group validity analysis was conducted, the significant differences of total MET-time scores were observed in certain subgroups (e.g., marital, education and employment status) suggesting an acceptable achieved discriminative validity for the self-reported PA questionnaire. However, no significant difference was found in the activity score by age, gender and BMI, which was similar with those found in the previous studies.^28,29^

The known group comparisons were not assessed in the level of subdomains which may be a reason for the non-discrepancy found in the results. As an evidence for this claim, the previous studies showed known groups discrepancy by relating subgroups to each domain of self-report PA.^15,30^ Despite these issues, it may indicate that the validity of the self-reported PA questionnaire is not influenced by age, gender, and BMI. As the participants were already engaged in the AZAR Cohort study (with Azari culture), this may limit the generalizability of our results to the general Iranian adults. However, the significant association of the MET-time score was not affected by age, gender, and BMI groups in the sample this limitation to be slight. Further research is warranted in a variety of settings, as there was not found any validation studies evaluating the self-reported PA questionnaire among adults with a cultural adaptation in Iran.

## Conclusion

The results of the present study suggested that the self-reported PA questionnaire has adequate psychometric properties for assessing PA in Khameneh adults. The modest reliability found for the instrument suggests that the self-reported PA questionnaire is internally consistent, stable, and valid. Although this instrument was applied in an Azari population in Iran, additional studies will be needed to better comprehend the psychometric properties of the scale among different populations.

## Acknowledgments

This research was conducted under a thesis grant for the Master’s degree from the Department of Statistics & Epidemiology, Tabriz University of Medical Sciences. We gratefully acknowledge the researchers of the AZAR Cohort Study for their close collaboration during the research process.

## Ethical approval

Ethics Committee in Tabriz University of Medical Sciences provided permission to conduct this survey. Signed informed consent was obtained from all participants of the AZAR Cohort Study prior to the data collection.

## Competing interests

The authors declare that there is no conflict of interest.

## Authors’ contributions

SD and NA-S contributed in original idea and protocol, conception of the work, conducting the study, revising the draft, approval of the final version of the manuscript, and agreed for all aspects of the work. MA-J contributed in the design of the work, doing the analysis, revising the draft and approval of the final version of the manuscript. FBA contributed in conception of the work and approval of the final version of the manuscript. MM contributed in conception of the work, conducting the study, wrote and editing of this manuscript.
